# Biopolymer Material from Human Spongiosa for Regenerative Medicine Application

**DOI:** 10.3390/polym14050941

**Published:** 2022-02-26

**Authors:** Ilya L. Tsiklin, Evgeniy I. Pugachev, Alexandr V. Kolsanov, Elena V. Timchenko, Violetta V. Boltovskaya, Pavel E. Timchenko, Larisa T. Volova

**Affiliations:** Biotechnology Center “Biotech”, Samara State Medical University, 443079 Samara, Russia; tsiklin.i@yandex.ru (I.L.T.); e.i.pugachev@samsmu.ru (E.I.P.); avkolsanov@mail.ru (A.V.K.); violetta.boltovskaya@yandex.ru (V.V.B.); timpavel@mail.ru (P.E.T.); volovalt@yandex.ru (L.T.V.)

**Keywords:** biopolymers, demineralized human spongiosa, scanning electron microscopy, micro-computed tomography, Raman spectroscopy, proteomic analysis, chondroblasts, tissue engineering, scaffold, fluorophores

## Abstract

Natural biopolymers demonstrate significant bone and connective tissue-engineering application efficiency. However, the quality of the biopolymer directly depends on microstructure and biochemical properties. This study aims to investigate the biocompatibility and microstructural properties of demineralized human spongiosa Lyoplast^®^ (Samara, Russian Federation). The graft’s microstructural and biochemical properties were analyzed by scanning electron microscopy (SEM), micro-computed tomography, Raman spectroscopy, and proteomic analysis. Furthermore, the cell adhesion property of the graft was evaluated using cell cultures and fluorescence microscopy. Microstructural analysis revealed the hierarchical porous structure of the graft with complete removal of the cellular debris and bone marrow components. Moreover, the proteomic analysis confirmed the preservation of collagen and extracellular proteins, stimulating and inhibiting cell adhesion, proliferation, and differentiation. We revealed the adhesion of chondroblast cell cultures in vitro without any evidence of cytotoxicity. According to the study results, demineralized human spongiosa Lyoplast^®^ can be effectively used as the bioactive scaffold for articular hyaline cartilage tissue engineering.

## 1. Introduction

Current treatment options for degenerative bone and cartilage tissue pathology aim to enhance post-traumatic and post-operative defects regeneration using various biological or synthetic products.

Biodegradable scaffolds, including calcium phosphate, aerogels [1,2,3,4,5,6], autologous [7,8,9], allogeneic [10,11,12], or xenogeneic grafts [13,14,15,16,17] demonstrate significant efficiency as bone substitute materials. Ideally, biocompatible materials’ resorption rate coincides with the formation rate of the new organotypic tissue. Allogeneic products incorporate identical structural and biological components and provide optimal conditions for genetically programed physiological regeneration in the human body [10,18]. Original technology of the human bone tissue products manufacturing developed at Samara Tissue Bank at Samara State Medical University has been successfully applied in bone tissue repair for more than twenty years. This technology provides thorough mechanical cleaning and complete removal of the antigenic components from human spongiosa while preserving its biological activity [18,19]. Microstructural and biochemical properties of the natural biopolymers play a crucial role in the regeneration process and directly depend on the manufacturing technology.

This study aims to investigate the microstructure and biocompatibility of the novel biopolymer material from demineralized human spongiosa.

## 2. Materials and Methods

### 2.1. Manufacturing and Characterizing Materials

**Figure 1:**
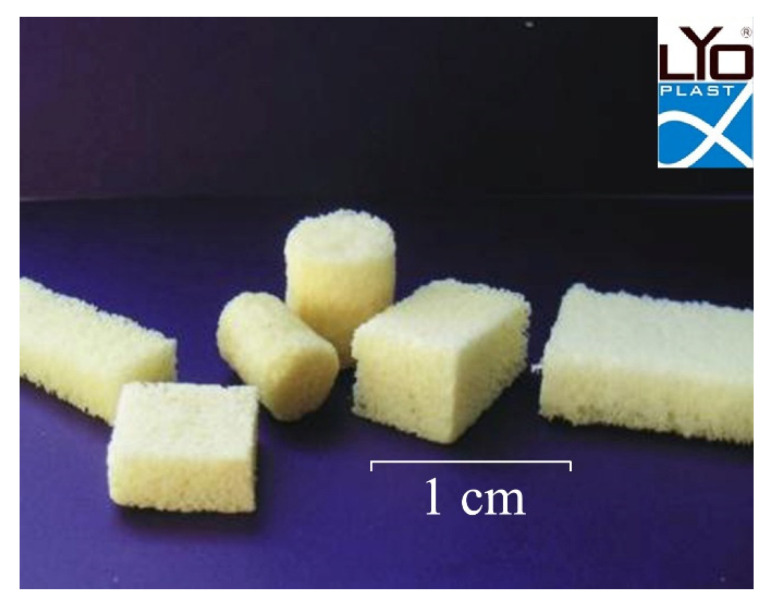
Samples of demineralized lyophilized human spongiosa Lyoplast^®^.

The biopolymer Lyoplast^®^ analyzed in this study is lyophilized demineralized human spongiosa manufactured at the Samara tissue bank at the “BioTech” Biotechnology Center, Samara State Medical University (RF patent No. 2366173 of 15.05.2008; certificate of conformity ISO 13485:2016, reg. No. RU CMS-RU.PT02.00115; certificate ISO 9001:2015, reg. No. TIC 15 100 159171) (Figure 1).

Experimental samples of Lyoplast^®^ material underwent compulsory low-frequency ultrasonic treatment using ultrasonic bath “Sapphire” TTC (RMD), (Sapphire LTD, Moscow, Russia with a frequency of 24–40 kHz.

Lyophilization of the material (vacuum drying by sublimation) was performed using a sublimation unit ALPHA2-4LSC (Martin Christ Gefriertrocknungsanlagen GmbH, Osterode am Harz, Germany).

Demineralization of human spongiosa was carried out in a weak HCl solution. Hermetically packaged lyophilized product was then sterilized with gamma rays using a certified GU-200 M (NIIP Joint-Stock Company, Moscow, Russia).

The residual content of lipids in the biomaterial was estimated using a spectrophotometer (SF-56 “Lomo-Spektr”, St. Petersburg, Russia). Finally, the humidity of the product was determined using a thermogravimetric infrared moisture meter (Sartorius-MA-150, Malente, Germany).

The study was carried out using physical, chemical, biological, and cultural methods.

### 2.2. Scanning Electron Microscopy (SEM)

The samples were examined using a JEOLJSM-6390 A Analysis Station SEM (Tokyo, Japan). Bioimplant samples were washed and fixed with a 2.5% aqueous solution of glutaric aldehyde. After that, they were spiked with ethanol of increasing concentration, followed by drying at room temperature for 24 h. Immediately before the study, the biomaterial was sprayed with gold or carbon to improve the surface electrical conductivity required for SEM.

### 2.3. Micro-Computed Tomography (Micro-CT)

Micro-CT scanning of the Lyoplast^®^ lyophilized allogeneic spongiosa samples was performed in Laboratory of Microanalysis in Skolkovo Technopark (Moscow, Russia) using high-resolution 3D X-ray microscope VersaXRM-500 (Xradia, Inc. Pleasanton, CA, USA) with voltage range 30–160 kV, maximum power 10 W, 360° rotation, and maximum spatial resolution < 0.7–1 µm (True Spatial Resolution™). At the first stage, the scanning of the sample was performed using a resolution of 8.6 µm/pixel at a voltage of 80 kV with a set of 1081 projections and 0.5 s exposition ti0me. Next, a region of interest (ROI), including bone trabeculae [20] was selected and scanned with a resolution of 1.1 μm/pixel at a voltage of 80 kV with 1441 projections and 0.5 s exposition time. The obtained data were reconstructed with the Filtered Back Projection method using the XRM Reconstructor software. Computed microtomography data were saved in TXRM and DICOM formats, and 3D models of the sample structure were saved in TXM and TIFF formats.

### 2.4. Raman Spectroscopy (Raman Spectroscopy)

This research was performed at the Department of Laser and Biotechnical Systems of Samara National Research University. Spectral characteristics of lyophilized, demineralized human spongiosa Lyoplast^®^ were studied using an experimental setup that included a high-resolution digital spectrometer Shamrock SR-303I (Oxford Instruments PLC, Abingdon, UK) with a built-in cooling chamber AndorDV420A-OE (Oxford Instruments PLC, Abingdon, UK) and an RPB785 fiber-optic probe combined with a laser module LuxxMasterLML-785.0RB-04(Laser Components Germany GmbH, Olching, Germany), all under the control of a PC workstation. This spectrograph provided 0.15 nm wavelength image resolution with low intrinsic noise. To exclude the autofluorescence contribution in the Raman spectra, we used a method for subtracting the fluorescence component of the polynomial approximation with additional filtration of random noise effects. In this work, the Raman spectra were analyzed in 350–2200 cm^−1^. The laser power of 400 mW was applied for 30 s exposure time, without evident degradation of the samples. Raman spectra were registered using an optical probe, which was placed above the object at a distance of 7 mm. We used the method of spectral contour fitting and deconvolution of the Gaussian function in the software environment MagicPlotPro 2.7.2. Thus, we conducted a non-linear regression analysis of Raman spectra to decompose the signal into spectral lines [21,22,23,24].

### 2.5. Mass Spectroscopy (Proteomic Analysis)

Samples of lyophilized, demineralized spongiosa were subjected to heat treatment (100 °C, 5 min) in a medium containing 2% SDS (sodium dodecyl sulfate) and 5% mercaptoethanol, centrifuged, and separated in a 10% polyacrylamide gel in the presence of SDS. After electrophoresis, the gel was stained with Coomassie G250 and cut into fragments according to the visually detectable fractions. For qualitative identification of tightly bound proteins, gel fragment samples were washed with ammonium hydrogen carbonate and acetonitrile solution (1:1) at 50 °C, dehydrated in 100% acetonitrile, and then treated with trypsin solution in 50 mM ammonium hydrogen carbonate. The reaction was stopped with the addition of 0.1% trifluoroacetic acid, and the released peptides were extracted from the gel by placement in an ultrasonic bath and separated by high-performance liquid chromatography (Dionex Ultimate 3000, Country) using an AcclaimPepMap C18 analytical column (2 μm, 100 Å, 75 μm × 15 cm) (Thermo Scientific). Mass spectra of the eluant were obtained on a maXis Impact mass spectrometer (Bruker, Germany) equipped with a CaptiveSpray (Bruker, Germany) ion source. The mass spectra were processed using DataAnalysis 4.1 software to obtain the mass lists, following a preset script to analyze continuous chromatograms. Proteins were identified from the mass lists using the Mascot 2.4.0 program (Matrix Science). Protein representation in the sample was evaluated using Multi Quant 3.0.2 software from the MRM transition peak area.

### 2.6. Obtaining a Line of Human Juvenile Chondroblasts

We used a culture of juvenile chondroblasts obtained from cartilage fragments of the interphalangeal joints of removed extra toes in healthy children diagnosed with polydactyly. Collection of biological material for cell culture was performed after parents/legal representatives had signed voluntary informed consent and with approval from the Bioethics Committee at Samara State Medical University. Tissue donors were somatically healthy and negative for HPV, HIV, HBsAg, and HCV infections. Material preparation was performed in the Biotech Department “BioTech” cell culture laboratory at Samara State Medical University. This laboratory is equipped with a suite of class B “clean rooms”, with the possibility of upgrading to class A areas following ISO 5 standard. Cartilage tissue fragments were washed three times with sterile Hanks’ solution, mechanically crushed, and then subjected to enzymatic treatment with 0.1% collagenase solution Biolot LLC (Saint Petersburg, Russia) for two hours in a shaker incubator BioSan (Riga, Latvia). The enzyme was inactivated by adding sterile 0.02% Versene solution (Biolot LLC, Russia). The material was transferred to centrifuge tubes and was washed in complete growth medium 199 containing 10% fetal calf serum (Biolot LLC, Russia) and spun at 1500 rpm on a low-temperature centrifuge Eppendorf 572 R (Eppendorf SE, Hamburg, Germany) at 4 °C for 20 min. The pellet was transferred into sterile plastic culture dishes (TRR Techno Plastic Products AG, Trasadingen, Switzerland) with an area of 25 cm^2^. A fresh portion of 10% 199 complete growth medium was added to the culture vial, then placed in a CO_2_ incubator. The condition of cells in the culture vial was monitored daily by observation under an inverted microscope Olympus CKX-41 (Olympus Corporation, Tokyo, Japan). The culture medium was changed every three days until the culture had attained 80% confluence, at which point the cells were transferred into new culture vials. The obtained chondroblast lines were analyzed for viability at the second passage using fluorescence microscopy.

### 2.7. Creation of a Cell-Tissue Graft

The Lyoplast^®^ tissue engineering product is a 3D carrier of demineralized human spongiosa seeded with juvenile chondroblasts. The chondroblasts were seeded on the scaffold. Cell culture was removed from the bottom of the culture plate at four passages in a traditional manner and seeded at a density of 5 × 104 cells per 27 mm^3^ (3 mm × 3 mm × 3 mm) block of the medium. The tissue-engineered constructs were placed in 7 mL vials with a complete growth medium (2 samples per vial) and were cultured in an incubator at 5% CO_2_ and 20% O_2_ at 37 °C. Samples of demineralized spongiosa Lyoplast^®^ of similar size but without chondroblasts served as a control group.

### 2.8. Fluorescence Microscopy

Fluorescence microscopy was performed using the Leica DMIL LED fluorescence module (Germany). For this purpose, a Live/Dead^®^ Viability/Cytotoxicity AssayKitfluorophore kit (Thermo Fisher Scientific Inc, Waltham, MA, USA) was used. Staining was performed according to the manufacturer’s protocol. The kit contains Calcein-AM and ethidium homodimer-1 solutions and is designed for simultaneous fluorescent staining of live and dead cells. Calcein-AM, the acetoxymethyl ester of calcein, is highly lipophilic and permeable to cell membranes. Although calcein-AM is not a fluorescent molecule, calcein produced by esterase reaction with calcein-AM in viable cells emits strong green fluorescence (490 nm excitation, 515 nm emission), while calcein-AM stains only viable cells. On the other hand, the nucleus staining dye ethidium homodimer-1 cannot pass through cell membranes, but it penetrates the membrane of dead cells, reaches the nucleus, and intercalates with the double helix of the cell DNA, where it shows red fluorescence (535 nm excitation, 617 nm emission). Thus, living cells glow green, while dead cells glow red.

### 2.9. MTT Assay for the Study of Demineralized Human Spongiosa Cytotoxicity

A culture of juvenile human chondroblasts of the seventh passage was used to study the cytotoxicity of the human spongiosa. A culture of human chondroblasts was seeded on a 24-well plate (SPL LIFE SCIENCES, Gyeonggi-do, Korea) at a dose of 4 × 10^4^ cells/well and incubated in a CO_2_ incubator Sanyo MSO-18AC (SANYO Electric Co., Ltd., Osaka, Japan) at 37 °C and 5% CO_2_. Control №1—medium only; control №2—medium with spongiosa. Probes №1 and №2 are cells only and cells with spongiosa, respectively. After the monolayer reached 80% confluency, the nutrient medium 199 Biolo t LLC, (Saint Petersburg, Russia) with a serum content of 10% (Biolo T, Russia) was changed in the wells, and Lyoplast^®^ spongiosa samples Lyocell (Samara, Russia) were placed in cubes 5 mm × 5 mm × 5 mm in size. Permeable plate inserts SPL LIFE SCIENCES, Gyeonggi-do, Korea were used to hold the material above the monolayer to exclude mechanical damage to the monolayer. After 48 h of cultivation with the material, cell viability was determined using 3-(4,5-dimethylthiazol-2-yl)-2, 5-diphenyltetrazolium bromide (MTT) (Sigma-Aldrich, Merck KGaA, Darmstadt, Germany), which allows evaluating the ability of live cells to convert soluble tetrazolium salt into an insoluble purple precipitate of formazan due to the action of cellular dehydrogenases. After removing the medium, each well was incubated with 0.3 mg/mL MTT in a growth medium at 37 °C for 3.5 h. At the end of the incubation period, the medium was removed by pipetting; intracellular formazan was dissolved in 200 μL of DMSO; and the optical density was measured at 550 nm on a Tecan Infinite M200 Pro microplate reader (Tecan Group Ltd., Männedorf, Switzerland). The percentage of viable cells was determined based on the calculated optical density, taking as 100% the values in the wells with cells without material.

## 3. Results and Discussion

### 3.1. Materials Characterization

Low-frequency ultrasound exposure, such as cavitation and microwaves, ensured complete destruction and removal of all cellular and bone marrow components, including bone cells, stroma, myeloid cells, and lipids from the spongiosa. Quality control of the scaffold delipidization was performed using physical and biochemical methods listed below. After lyophilization in a sublimation unit, the humidity of the biomaterial did not exceed 5%.

Exposure of the material to hydrochloric acid resulted in the final destruction of bone cells and demineralization of the scaffold.

The shelf life of lyophilized demineralized spongiosa Lyoplast^®^ is three years after gamma-ray sterilization. Storage and transportation of the finished product does not require any special temperature conditions.

### 3.2. Scanning Electron Microscopy

**Figure 2:**
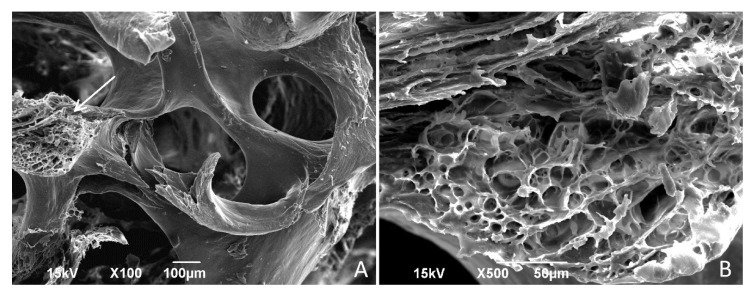
Architectonics of demineralized lyophilized human spongiosa (SEM). Trabecular architecture: (**A**) cross section of trabeculae (marked by an arrow), ×100; internal structure of the trabeculae (**B**) ×500.

SEM examination of human spongiosa that underwent all stages of the Lyoplast^®^ bioimplant production process, including ultrasound treatment of the biomaterial, demineralization, lyophilization, and γ-sterilization, showed complete preservation of the original trabecular bone architectonics. According to the results of the SEM, the completed biopolymer product is a porous three-dimensional (3D) matrix with a hierarchical structure of pores of various calibers (300–800 µm), free of cellular and bone marrow components. Trabeculae of lamellar bone are visualized on the image; their contours are precise and interconnected to form fine pores. Similarly, these fine pores do not contain cellular and bone marrow components. The 3D photos demonstrate a self-similar hierarchical architecture of the human spongiosa organization (Figure 2).

### 3.3. Micro-Computed Tomography

**Figure 3:**
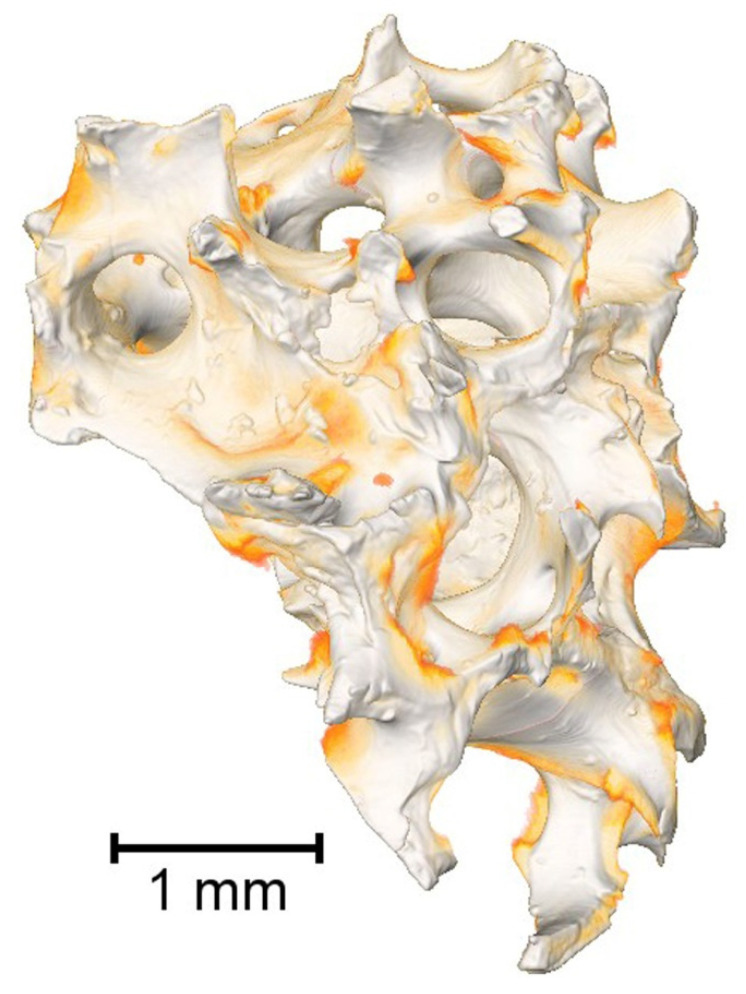
Architectonics of demineralized lyophilized human spongiosa (Micro-CT). Trabecular architecture: (80 kV, 8.6 μm/pixel; 1081 projections; 0.5 s exposition time).

Non-destructive microstructural analysis of the Lyoplast^®^ human spongiosa bioimplant using computed microtomography confirmed the preservation of spongy bone’s trabecular architectonics and porous microstructure with a pore caliber of 300 to 800 µm. Three-dimensional reconstruction of bone tissue samples using a resolution of 8.6 μm/pixel and a voltage of 80 kV made it possible to visualize the surface of the pores, free from cellular debris and fatty components of the bone marrow (Figure 3).

**Figure 4:**
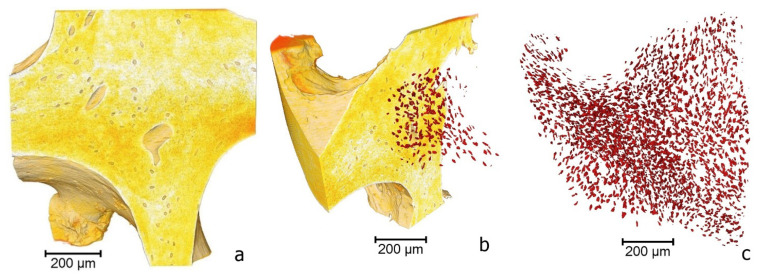
Architectonics of demineralized lyophilized human spongiosa (Micro-CT). Trabecular architecture: (80 kV, 8.6 μm/pixel; 1081 projections; 0.5 s exposition time) (**a**)—visualization of the osteocyte lacunae; (**b**,**c**)—image inversion and osteocyte lacunae detection).

Using a resolution of 1.1 μm/pixel and a voltage of 80 kV confirmed the hierarchical bone tissue architectonics and allowed us to visualize osteocyte lacunae in the intrinsic structure of bone trabeculae. The average caliber of osteocyte lacunae was 10–30 μm. The investigation also confirmed the absence of a cellular component in the lacunae. In addition, three-dimensional image inversion allowed a detailed analysis of the number and condition of osteocyte lacunae. (Figure 4). An essential advantage of micro-computed tomography compared to scanning electron microscopy (SEM) is the combination of high-throughput fashion and ultra-high resolution, making it possible to simultaneously visualize a significant number of osteocyte lacunae while maintaining high image quality.

### 3.4. Raman Spectroscopy

**Figure 5:**
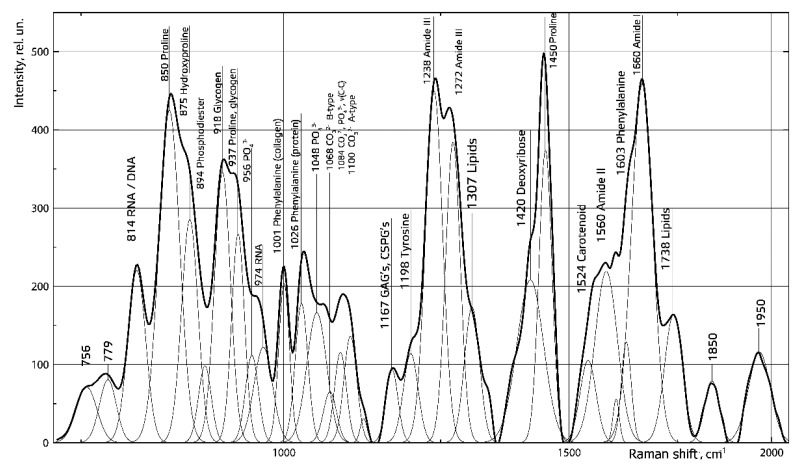
Spectral contour decomposition of demineralized spongiosa samples. The solid line-original Raman spectrum; the dashed lines-the Raman lines after separation.

Raman spectroscopy allowed us to obtain detailed information about spectral contour decomposition of demineralized spongiosa samples using a Gaussian function as a trial (Figure 5). The mean value of the coefficient of correlation between the recovered and input spectrum (R2) in the region of 750–2050 cm^−1^ was 0.99, indicating near-perfect agreement.

We have established no mineral components in the demineralized spongiosa, as indicated by the disappearance of the Raman line at 956 cm^−1^, corresponding to 𝑃𝑂43−(𝑣1). As can be seen in Figure 3, the demineralized spongiosa lacks fat, as indicated by the absence of an intense Raman line at 1307 cm^−1^ (lipids). At the same time, the preservation of the organic matrix is observed, as indicated by the presence of Raman lines at 850 cm^−1^ (proline), 1238 cm^−1^–1272 cm^−1^ (Amide III), 1450 cm^−1^ (proline), 1167 cm^−1^ (glycosaminoglycans), and 1660 11 cm^−1^ (Amide I). Collagen is the main protein component of bone tissue, and it forms the fibrillar framework of the bone matrix. The amino acid sequence of collagen is notably rich in proline, about half of which is hydroxylated during collagen breakdown to form hydroxyproline.

### 3.5. Mass Spectroscopy

Proteomic analysis of the demineralized human spongiosa demonstrated the presence of collagenous and extracellular bone matrix proteins. The ability of these proteins to stimulate and inhibit cell adhesion, proliferation, and differentiation is noteworthy. We identified five main types of collagen (I, IV, VI, XII, XIV), fibronectin, vitronectin, osteopontin, matrix Gla-protein, along with TGF-β mimecan, decorin, and other proteins in the human spongiosa organic matrix. A list of these proteins, their localization, and their mass are presented in Table 1.

We assign properties and functions of the identified proteins according to Baghy et al. [25,26,27,28]. In summary, collagen acts as a cell-binding protein that performs an adhesive function by integrating collagen bundles, a major component of the extracellular matrix. Bone, basal membrane, and soft tissue collagens are found in human spongiosa. Fibronectins bind cell surfaces and compounds, including collagen, fibrin, heparin, DNA, and actin. Fibronectins are involved in cell adhesion, cell motility, opsonization, wound healing, and maintenance of cell shape. They also participate in the regulation of type I collagen deposition by osteoblasts. Vitronectin is a glycoprotein of the hemopexin family, which is abundant in serum and the extracellular matrix of bone tissue. It is involved in fibrinolysis, mediates cell adhesion and migration, and binds glycosaminoglycans, collagen, and plasminogen. These three organic substances (collagen, fibronectin, vitronectin) are widely used in biotechnology to create the cytoadhesive surface of the culture plate.

Mimecan, or osteoglycin, is a small proteoglycan rich in leucine, important for collagen fibrillogenesis. Decorin, lumican, and biglican are small proteoglycans of the extracellular matrix that bind to fibronectin, inhibit cell adhesion, attach to tumor growth factor, and reduce tumor cells’ mitogenic activity. They play a regulatory role in connective tissue development and repair processes. TGF-β1 is a secreted protein that performs many cellular functions, including control of cell growth, cell proliferation, cell differentiation, and apoptosis. Osteopontin is involved in cell proliferation, migration, and adhesion, including bone marrow mesenchymal stem cells, hematopoietic stem cells, osteoclasts, and osteoblasts. Osteonectin is a bone tissue glycoprotein that binds calcium. It is released by osteoblasts during bone formation, initiating mineralization and promoting the formation of mineral crystals. Osteonectin also has an affinity for collagen. Finally, bone sialoprotein is a critical component with a high sialic acid content of the extracellular bone matrix, constituting approximately 8% of all non-collagenous proteins. It has the function of forming the hydroxyapatite core during bone mineralization. Matrix Gla-protein associates with the organic matrix of bone and cartilage and acts as an inhibitor of bone formation, thereby playing an essential role in bone mineralization but acting as a mineralization inhibitor in cartilage and vessels. Finally, the extracellular matrix protein tenascin is involved in the control of migrating neurons and axons during neuronal development, synaptic plasticity, and regeneration. Its role in osteoblasts differentiation is unknown. This comprehensive analysis of protein constituents complements the results of Raman spectroscopy and provides a broader understanding of the biochemical composition of the organic matter of human spongiosa Lyoplast^®^.

### 3.6. Obtaining a Line of Human Juvenile Chondroblasts and Creating a Cell-Tissue Graft

Observations of the native culture showed that cells had excellent adhesive properties.

On the first day in culture, most chondroblasts from the suspension were deposited from the medium to the bottom of the plate, where they attached and spread out. They took on an elongated shape with a well-defined boundary, connecting through 3–5 processes. The cytoplasm contained many vacuoles in the peripheral zone. The nucleus was oval shaped, usually located in the center of the cell, and contained 1–3 nuclei. The number of cells gradually increased during cultivation, forming a uniform monolayer. When the culture reached 80% confluence, the chondroblasts adhered tightly to each other, and there were practically no areas free of cells. At this stage, cell transplantation was performed using the standard method. A qualitative culture assessment was carried out using cultural and morphological methods in the fourth passage. Our earlier preclinical animal studies, using combined cell-tissue grafts based on rabbit allogenic demineralized spongiosa and rabbit rib cartilage cell culture, showed the healing of the animal joint’s simulated bone and cartilage defects. Finally, organotypic hyaline cartilage tissue was revealed [19], and it is promising for translational use of Lyoplast^®^ human spongiosa. The unique structure and composition of the demineralized human spongiosa allowed us to use it as an effective bioscaffold for creating the cell-tissue graft.

The findings reported by Doran and his study group members in 2021 [29] also demonstrated the efficiency of using cartilaginous differon cells for articular cartilage tissue restoration. Obtained results demonstrate the prospect for further use of demineralized human spongiosa for articular hyaline cartilage defects repair. Obtaining such cell-tissue grafts is relatively simple and does not require complex bioreactor systems.

**Figure 6:**
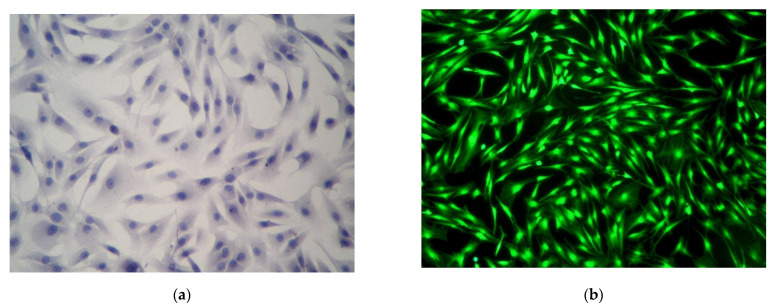
Human juvenile chondroblast culture: (**a**) hematoxylin and eosin staining. Light microscopy, ×100; (**b**) live chondroblasts exhibiting bright green glow. Fluorescence microscopy. Staining with Live/Dead^®^ fluorophores, ×100.

Cell cultures stained with hematoxylin and eosin showed the geometric pattern typical of cartilage differon cells. The cells were aligned, forming concentric and ellipsoidal figures resembling osteons and insertion plates of compact bone tissue. The presence of polygonal cells was noted. The cytolemma of chondroblasts in culture was smooth, and the weakly oxyphilic cytoplasm contained vacuoles. Nuclei of regular round shape with a smooth envelope were located mainly in the center. Chromatin in the form of fine granularity was located diffusely in the nuclei. The large processes were shortened, and the intercellular substance was visualized as translucent layers (Figure 6a). The fluorescence microscopy with a Live/Dead^®^ fluorophore kit revealed 93% viable cells (Figure 6b).

**Figure 7:**
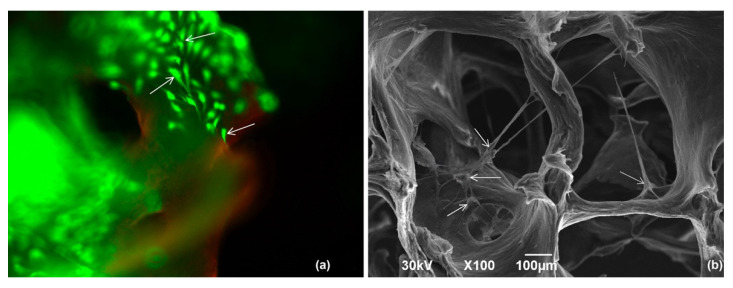
Demineralized spongiosa using the Lyoplast^®^ technology with populated chondroblasts: (**a**) populated viable chondroblasts on the surface of the biocarrier (arrows indicate attached cells). Live/Dead^®^ fluorophore staining. Fluorescence microscopy, ×100; (**b**) cells inhabiting the surface of trabeculae are marked by arrows. SEM, ×100.

Upon examining living and damaged cells populated on a 3D carrier made of Lyoplast^®^ human spongiosa using the Live/Dead^®^ kit, we detected viable cells with bright green coloration and damaged or dead cells with a bright red nucleus (Figure 7a). On closer examination, the cells on the surface of the trabeculae were evenly distributed throughout the scaffold depth, and the shape was polygonal (elongated, triangular, rounded). We visualized the outgrowths with which the chondroblasts were connected, creating a uniform monolayer, which was indicative of good cell adhesion to this material. When the cell-tissue graft was examined using SEM, we saw adsorption of proteins on the trabeculae surface, where cells of elongated shape were connected by outgrowths (Figure 7b).

### 3.7. MTT Assay of the Demineralized Human Spongiosa MTT Test Results

**Figure 8:**
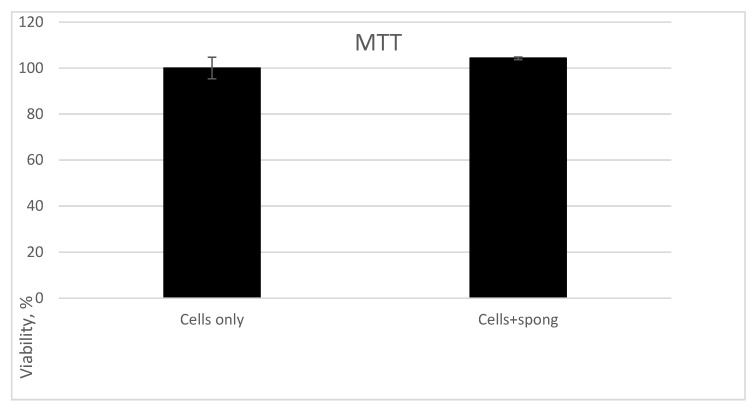
MTT test. No difference in cell viability between control (Cells only) and probe (Cells + Spong) was detected by MTT test.

MTT Assay of the Demineralized Human Spongiosa MTT Test Results Are Presented in Table 2 and Figure 8.

Using MTT test (48 h treatment), it was found that for the experimental group (Cells + Spong and controls (Cells only)), the optical density was 0.491 ± 0.023 and 0.512 ± 0.003, correspondingly. Statistical analyses revealed that no significant statistical differences in cell viability (*p* = 0.513) were observed between control cells (Cells only) and cells grown in the presence of the investigated biomaterial (Cells + Spong).

Thus, the investigated biomaterial—demineralized human spongiosa Lyoplast^®^—is not cytotoxic.

## 4. Conclusions

Microstructural analysis of the demineralized human spongiosa convincingly demonstrates its preserved hierarchical porous structure. Pores of various configurations and calibers are entirely free of cellular debris and bone marrow components. Furthermore, the proteomic analysis confirmed the complete preservation of collagen and other extracellular matrix proteins. These proteins play a crucial role in inhibiting and stimulating cell adhesion, proliferation, and differentiation. We revealed the adhesion of chondroblast cell cultures in vitro without any evidence of cytotoxicity. Revealed features of the demineralized human spongiosa create optimal conditions for hyaline articular cartilage and subchondral bone regeneration. Thus, according to the study results, demineralized human spongiosa Lyoplast^®^ can be effectively used as the bioactive scaffold for articular hyaline cartilage tissue engineering. For the future perspective, the identified microstructural and biochemical features of the demineralized human spongiosa can be considered in 3D bioprinting and creating tissue-engineering constructs of the cartilage tissue.

## 5. Patents

Patent RF № 2170016 from 17.02.1999 “Method of saturation of bone spongy tissue grafts with medication” Volova L.T., Kirilenko A.G., Uvarovsky B.B.

Patent RF № 2156139 from 15.03.1999 “Method of sterilization of lyophilized bone transplants” Volova L.T., Kirilenko A.G., Uvarovsky B.B.

Patent RF № 99108699 from 21.04.1999 “Method of bone marrow removal from cancellous bone grafts” Volova L.T., Kirilenko A.G.

Patent RF № 2366173 of 15.05.2008. “Method of manufacturing large-block lyophilized bone implants” Volova L.T.

## Figures and Tables

**Table 1:** List of organic components identified in the analysis of demineralized human spongiosa Lyoplast^®^.

Proteins (Polypeptide Chains)	Localization	Mass, kDa
Matrix Gla-protein	Bone	12,353
Secreted phosphoprotein 24	Bone	24,338
Transforming growth factor beta-1 (TGF-β1)	Bone	25,000
Mimecan	Bone	33,922
SPARC (Osteonectin)	Bone	34,632
Bone sialoprotein 2	Bone	35,148
Osteopontin	Bone	35,423
Lumican	Extracellular matrix (ECM)	38,429
Decorin	ECM	39,747
Chondroadherin	Cartilage matrix protein	40,476
Biglycan	ECM	41,654
Fibromodulin	ECM	43,179
Prolargin	ECM	43,810
Osteomodulin	Bone	49,492
Vitronectin	Plasma, ECM	75,000
Collagen (I) alpha chain	ECM	108–168
Collagen (IV) alpha chain	ECM	108–168
Collagen (VI) alpha chain	ECM	108–168
Collagen (XII) alpha chain	ECM	108–168
Collagen (XIV) alpha chain	ECM	108–168
Tenascin	ECM	240,853
Fibronectin	Plasma, ECM	262,625

**Table 2:** MTT test. Indicators of optical density in experimental and control wells.

	Controls		Probes	
	Medium	Medium + Spong	Cells	Cells + Spong
**OD, units**	0.0716	0.0782	0.491	0.512
**STDV**	0.007163155	0.000260349	0.023076494	0.002760717

## Data Availability

Data available on request due to restrictions eg privacy and ethical.
